# False, Reversed but Not True: A Curious Case of Hyperkalemia

**DOI:** 10.7759/cureus.10066

**Published:** 2020-08-27

**Authors:** Munnam S Jafar, Nishanth Thalambedu, Lakshmi Kolandra, Sohaib Roomi

**Affiliations:** 1 Internal Medicine, Abington Hospital - Jefferson Health, Abington, USA

**Keywords:** renal medicine, oncology, hyperkalemia, chronic lymphocytic leukemia, electrolyte disturbances, pseudohyperkalemia

## Abstract

Falsely elevated potassium levels are common in routine laboratory tests and should be differentiated from true hyperkalemia. If the patient is inappropriately treated for hyperkalemia, the resulting hypokalemia can lead to life-threatening cardiac arrhythmias. We present the case of a 67-year-old woman with a past medical history of stable chronic lymphocytic leukemia, who presented for chest pain and had an elevated potassium level of 5.8 mEq/L, which, upon repeat laboratory testing, was then 6.7 mEq/L. She was initially treated for hyperkalemia. Laboratory test results showed creatine kinase levels at 43 U/L, lactate dehydrogenase levels at 177 U/L, phosphorus levels at 4.5 mg/dL, and uric acid levels at 6.4 mg/dL, indicating no evidence of tumor lysis syndrome. The patient was later diagnosed with reverse pseudohyperkalemia, indicated by falsely elevated plasma potassium levels in the presence of serum potassium levels within normal limits and venous blood gas samples.

## Introduction

Falsely elevated potassium levels are commonly seen in routine laboratory tests and physicians should always differentiate false elevated potassium levels from true hyperkalemia. If the distinction is not made and the patient is treated for hyperkalemia, the resulting hypokalemia can lead to life-threatening cardiac arrhythmias [1,2].

## Case presentation

**Figure 1:**
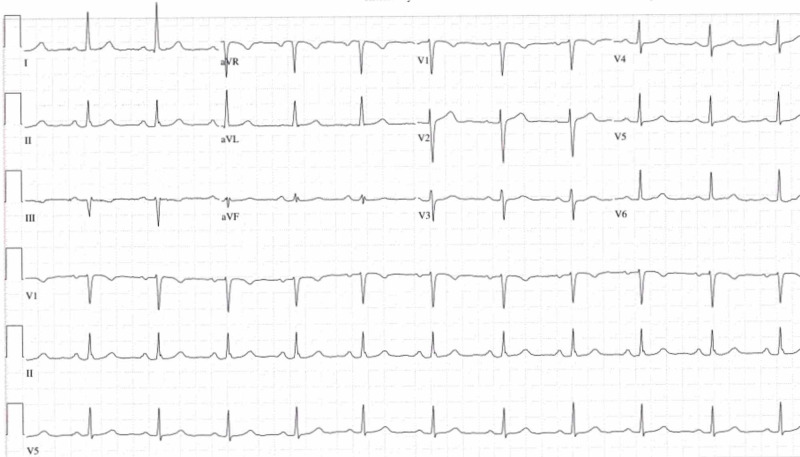
Electrocardiogram (ECG)

We present a case of a 67-year-old woman with a past medical history of stable chronic lymphocytic leukemia (CLL) for seven years who presented to the emergency department (ED) with chest pain. She was found to have an elevated potassium level of 5.8 mEq/L. The patient had an otherwise unremarkable past medical history and was not taking any medications. Her most recent outpatient white blood cell (WBC) count was 161,000/µL with lymphocytic predominance, which was stable when compared to her previous laboratory results. Repeat laboratory tests in the ED showed serum potassium levels at 6.7 mEq/L and creatinine levels at 0.9 mEq/L without any associated (ECG) changes (Figure 1). The patient was treated for hyperkalemia with calcium gluconate, insulin, and dextrose cocktail in the ED.

**Figure 2:**
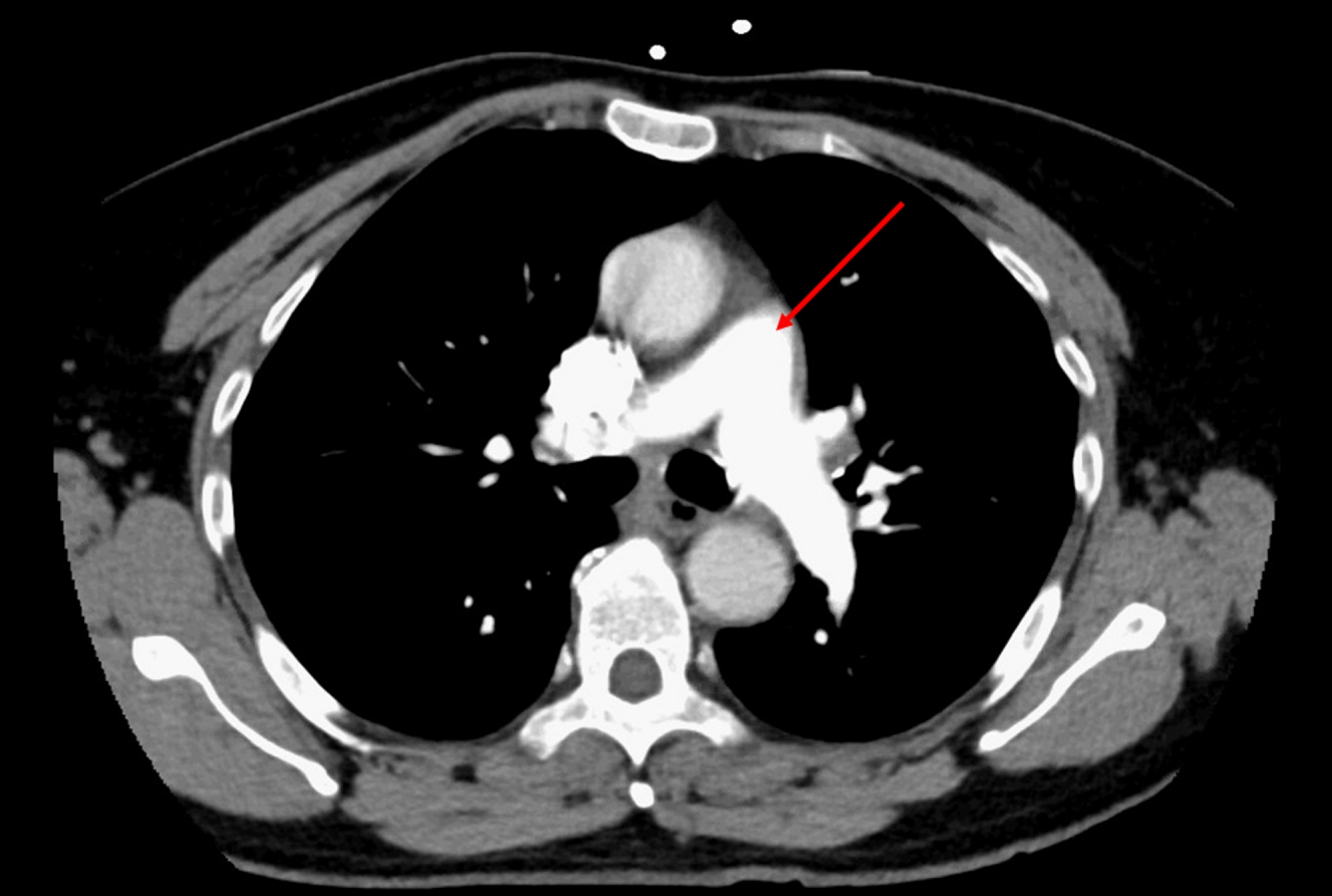
CT scan with pulmonary embolism protocol The arrow marks main pulmonary artery dividing into right and left branches. Lack of filling defect indicates the absence of pulmonary embolism.

Our patient had an unremarkable chest CT scan with pulmonary embolism protocol in the ED (Figure 2).

Her cardiac troponin trended negatively over the following 12 hours, and her chest pain was attributed to musculoskeletal etiology. The patient confirmed she was not taking any medicines or herbal supplements at home. Her CLL was being managed expectantly, and she had never received chemotherapy in the past. To determine the causes of hyperkalemia, further workup for tumor lysis syndrome revealed a creatine kinase level of 43 U/L, lactate dehydrogenase level of 177 U/L, phosphorus level of 4.5 mg/dL, and uric acid level of 6.4 mg/dL, effectively ruling out the condition.

This was followed by a detailed evaluation for hyperkalemia, which included comparing plasma potassium levels - with and without centrifugation - to serum and venous blood gas levels. Serum and venous blood gas potassium results were 4.0 mEq/L and 4.3 mEq/L, respectively. These levels were significantly different from plasma levels, which were 5.6 mEq/L without centrifugation and 5.8 mEq/L with centrifugation. We recognized this laboratory phenomenon and made a diagnosis of reverse pseudohyperkalemia in the setting of CLL. The patient was subsequently discharged to home and advised regular follow-ups with her oncologist. We also recognized that in our hospital, the ED uses plasma samples while the general medical floors use serum samples for processing blood electrolytes.

## Discussion

Pseudohyperkalemia, as is implicit in the name, is characterized by a spurious elevation in potassium levels. However, there are subtle details that healthcare providers must notice to differentiate the condition from a closely related clinical entity called reverse pseudohyperkalemia.

Serum is the residual portion of the blood left after clotting. Plasma is obtained when blood is not allowed to clot, which is accomplished by mixing it with anticoagulants such as heparin. Plasma without clotting factors is serum. Blood samples treated with heparin (plasma) are used routinely in hospital laboratory testing as the blood samples can be separated via centrifuge immediately upon arrival [1,2]. Pseudohyperkalemia is characterized by falsely elevated potassium levels in both blood serum and plasma. Reverse pseudohyperkalemia, alternatively, is elevated plasma potassium in the presence of typical serum values.

The etiology of pseudohyperkalemia is manifold. It is often associated with routine blood draws, for example, prolonged tourniquet time, traumatic venipuncture, excessive suctioning, vigorous handling of the blood sample, and extended processing time [2]. Moreover, potassium release from platelet degranulation can result in pseudohyperkalemia, particularly in patients with significant thrombocytosis [3].

Reverse pseudohyperkalemia, on the contrary, is a relatively rare and poorly understood laboratory phenomenon associated with hematological malignancies. Malignant cells are fragile and prone to lysis, especially with pneumatic tubes and vigorous sample handling during routine transport [4,5]. During centrifugation of samples with a very high WBC count, a layer of cells can be visualized in the plasma tubes above the gel, which demonstrates the breakdown of fragile malignant cells and corresponds with hyperkalemia [1]. Another possible explanation of this in vitro phenomenon is a high metabolic fuel demand by a greater number of malignant cells, causing depletion of adenosine triphosphate (ATP), which leads to dysfunction of the sodium-potassium ATPase pump system that normally helps keep potassium intracellular [2].

Heparin has also been implicated to play a role in reverse pseudohyperkalemia, as heparinized samples (plasma) have been shown to have elevated potassium levels when compared to corresponding non-heparinized samples (serum). Samples containing more heparin had elevated lactate dehydrogenase indicating possible lysis of red blood cells or malignant white cells in the presence of heparin [4]. The fibrin mesh around the clotted blood in serum samples may prevent fragile malignant cells from lysis [1]. Moreover, potassium values in arterial samples may be more accurate than in venous samples due to shorter processing time and less exposure to mechanical stressors [6].

## Conclusions

Reverse pseudohyperkalemia should be suspected in patients with CLL and an elevated WBC count. We recommend that high potassium levels in patients with elevated WBC levels should be flagged by laboratories. A healthy ECG in the proper clinical setting can assist in making the diagnosis at bedside. Checking serum and arterial/venous blood gas samples in patients with suspected reverse pseudohyperkalemia can help differentiate the condition from normokalemia and true, life-threatening hyperkalemia. Knowledge about this clinical entity is important because treating falsely elevated potassium levels can be fatal.
